# Recombinant lipoprotein-based vaccine candidates against *C. difficile* infections

**DOI:** 10.1186/s12929-015-0171-x

**Published:** 2015-08-07

**Authors:** Jui-Hsin Huang, Chia-Wei Wu, Shu-Pei Lien, Chih-Hsiang Leng, Kuang-Nan Hsiao, Shih-Jen Liu, Hsin-Wei Chen, Leung-Kei Siu, Pele Chong

**Affiliations:** Vaccine R&D Center, National Institute of Infectious Diseases and Vaccinology, National Health Research Institutes, Zhunan Town, Miaoli County Taiwan; Graduate Institute of Life Science, National Defense Medical Center, Taipei, Taiwan; Graduate Institute of Immunology, China Medical University, Taichung, Taiwan

**Keywords:** Antibiotic-associated pseudo-membranous colitis, *C. difficile* toxins, Receptor binding domain, Lipoprotein, Toll-like receptor agonist, Adjuvant

## Abstract

**Background:**

Opportunistically nosocomial infections in hospitalized patients are often related to *Clostridium difficile* infections (CDI) due to disruption of the intestinal micro-flora by antibiotic therapies during hospitalization. Clostridial exotoxins A and B (TcdA and TcdB) specifically bind to unknown glycoprotein(s) in the host intestine, disrupt the intestinal barrier leading to acute inflammation and diarrhea. The C-terminal receptor binding domain of TcdA (A-rRBD) has been shown to elicit antibody responses that neutralize TcdA toxicity in Vero cell cytotoxicity assays, but not effectively protect hamsters against a lethal dose challenge of *C. difficile* spores. To develop an effective recombinant subunit vaccine against CDI, A-rRBD was lipidated (rlipoA-RBD) as a rational design to contain an intrinsic adjuvant, a toll-like receptor 2 agonist and expressed in *Escherichia coli*.

**Results:**

The purified rlipoA-RBD was characterized immunologically and found to have the following properties: (a) mice, hamsters and rabbits vaccinated with 3 μg of rlipoA-RBD produced strong antibody responses that neutralized TcdA toxicity in Vero cell cytotoxicity assays; furthermore, the neutralization titer was comparable to those obtained from antisera immunized either with 10 μg of TcdA toxoid or 30 μg of A-rRBD; (b) rlipoA-RBD elicited immune responses and protected mice from TcdA challenge, but offered insignificant protection (10 to 20 %) against *C. difficile* spores challenge in hamster models; (c) only rlipoA-RBD formulated with B-rRBD consistently confers protection (90 to 100 %) in the hamster challenge model; and (d) rlipoA-RBD was found to be 10-fold more potent than A-rRBD as an adjuvant to enhancing immune responses against a poor antigen such as ovalbumin.

**Conclusion:**

These results indicate that rlipoA-RBD formulated with B-rRBD could be an excellent vaccine candidate for preclinical studies and future clinical trials.

## Background

*Clostridium difficile* has become an emerging infectious pathogen that is responsible for opportunistic infections in hospitals worldwide and is the main cause of antibiotic-associated pseudo-membranous colitis in humans [1–3]. Moreover, the finding of a hyper-virulent and antibiotic-resistant epidemic strain, BI/NAP1/027 in developed countries, poses a major challenge for preventing *C. difficile* infections (CDIs) [4–6]. The pathogenicity of *C. difficile* is largely mediated by two clostridial toxins, toxin A and toxin B (TcdA and TcdB), that are secreted in the gastrointestinal environment of infected hosts and disrupt the epithelial cell barriers in the small intestine [7]. Both toxins consist of holotoxins with multi-functional domains that mediate *C. difficile* pathogenesis. The mechanism underlying TcdA and TcdB toxicity involves three steps: (a) binding to unidentified receptor protein(s) on the surface of intestinal epithelium and internalization through its C-terminal receptor binding domain, (b) auto-cleavage and translocation of the N-terminal glucosyltransferase domain to the cytosol from the endosomal membrane; and (c) the N-terminal enzymatic region that inactivates the Rho GTPase family by glycosylation [7, 8].

Interestingly, TcdA-specific antibodies in patient sera were found to positively correlate with the prevention of CDAD recurrence [9–12]. Therefore, passive immunization with anti-toxin antibodies has been shown to confer protection against CDI in animal models and TcdA-specific monoclonal antibodies are currently being tested in clinical trials [13–15]. In addition, different *C. difficile* vaccine strategies are being evaluated; the most advanced being vaccination with formalin-inactivated toxins [16–19]. Immunization with the receptor binding domain (RBD) of *C. difficile* toxins as an antigen formulated with different adjuvants has been shown to elicit toxin-neutralizing antibody responses and protect mice from toxin or bacteria challenges [20–26]. TcdA RBD (A-rRBD) has a molecular size around 100 kDa and is composed of 32–38 homologous repetitive peptides which contain 7 potential lectin-like receptor-binding sites for binding to the synthetic oligosaccharide, Galα1-3Galβ1-4GlcNAc that is often found in glycoproteins [8, 20, 27–29]. The specific roles and functions of these 7 putative binding regions are unclear. In our previous study [30], a recombinant A-rRBD based on the consensus sequence of TcdA identified from different *C. difficile* strains obtained from the NCBI protein database and three truncated fragments of RBD corresponding to the N-terminal (residues 1–411), middle (residues 296–701), and C-terminal parts (residues 524–911) (F1, F2 and F3, respectively) were designed and expressed in *E. coli*. The purified A-rRBD and its fragments were characterized biologically and found to have the following properties: (a) A-rRBD and the truncated fragments are capable of binding to the cell surface and internalizing into both Vero and Caco-2 cells; (b) A-rRBD, F3 and F2 show various level of hemagglutinin (HA) activity, but F1 has no HA activity; and (c) A-rRBD and the truncated fragments can act as a toll-like receptor agonist activating dendritic cell maturation, but F3 is the most potent. The results indicated that F1, F2 and F3 have similar repetitive amino acid sequences and putative oligosaccharide-binding domains, but they do not express the same level of biological properties. In another study [31], a TcdB RBD derived from *C. difficile* strain VPI10463 which has >95 % amino acid sequence identity to BI/NAP1/027 hyper-virulent strains was designed and expressed in *E. coli*. Recombinant TcdB RBD (B-rRBD) was purified, characterized biologically and immunologically, and found to have the following properties: (a) capable of binding to the cell surface of both Vero and Caco-2 cells and entering into the cytosol; (b) showing no hemagglutinin activity (HA); (c) functioning as a toll-like receptor agonist activating dendritic cell maturation; (d) in the absence of adjuvant, eliciting anti-TcdB neutralizing antibody responses that could weakly cross-neutralize TcdA; and (e) inducing partial protection against a lethal dose of *C. difficile* spores in the hamsters challenge model. To develop an effective recombinant subunit vaccine against CDI, in this study, A-rRBD was lipidated (rlipoA-RBD) as a rational design to contain an intrinsic adjuvant, toll-like receptor 2 agonist and expressed in *E. coli*. The purified rlipoA-RBD was further characterized immunologically and tested to determine whether it could be a highly efficacious vaccine candidate against CDAD, or if it required formulation with B-rRBD and adjuvant.

## Methods

### Ethics statement

All experiments were conducted in accordance with the guidelines of the Laboratory Animal Center of National Health Research Institutes (NHRI). Animal use protocols have been reviewed and approved by the Institutional Animal Care and Use Committee of National Health Research Institutes (Approved protocol No. NHRI-IACUC-100053-A).

### Production of A-rRBD and B-rRBD

The purification of A-rRBD and B-rRBD expressed in *E. coli* JM109 (DE3) strain have been previously described [30, 31]. All purification steps were analyzed by 8 % SDS-PAGE. The residual endotoxin was determined using the Limulus amoebocyte lysate (LAL) assay (Associates of Cape Cod, Inc., Cape Cod, MA).

### Construction, expression and purification of rlipoA-RBD

Construction of plasmid containing rlipoA-RBD was cloned into the pET-22b (+) vector using Bam HI and Xho I sites as previously described [32]. These constructs were expressed in the *E. coli* C43 (DE3) strain. In brief, the 3’-end of A-rRBD was fused with the sequence containing a polyhistidine tag and XhoI restriction enzyme site [30]. The 5’ terminus was fused to *E. coli.* lipidated signal sequence by BamHI restriction enzyme site [32]. The 5’-end of lipid leader sequence also contained a NdeI restriction enzyme site. Finally, A-rRBD nucleotide sequence possessing 5’-lipid leader sequence and 3’ polyhistidine sequence containing NdeI and XhoI sites, respectively, was cloned into pET-22b(+) vector (Novagen, Darmstadt, Germany) by the NdeI and XhoI restriction enzyme sites. This pET-22b(+)_rlipoA-RBD construct was transformed into *E. coli* C43 (DE3) (Imaxio; Saint-Beauzire, France) for rlipoA-RBD expression. rlipoA-RBD was over-expressed in 5 liters of LB Broth containing 100 μg/ml ampicillin by *E. coli* C43 (DE3) (Imaxio; Saint-Beauzire, France). Once OD_600nm_ of bacteria culture achieved approximately 0.5, 1 mM isopropyl-β-D- thiogalacto-pyranoside (IPTG) was added into the culture medium to incubate at 20 °C for 16 h. Bacteria were harvested by centrifugation and stored at −20 °C before lysis. Bacterial pellet was suspended in lysis buffer (50 mM Tris-Cl, pH8.0 containing 500 mM NaCl) and disrupted physically by French Press (Constant System, Daventry, UK) at 27 Kpsi. Cell lysate was pelleted and extracted twice with 50 mM Tris-Cl, pH8.0 containing 0.5 % Triton X-100. The crude-extracted solution was purified by two step affinity chromatograph. First, nickel resin was used to separate any impurities. The eluent was dialyzed to remove imidazol and applied to an immobilized metal affinity chromatography (IMAC) (GE Healthcare, Uppsala, Sweden) charged with copper ion for LPS removal. All purification steps were performed at 4 °C and analyzed by 8 % SDS-PAGE. Affinity chromatography was performed according to manufacturer’s instruction. The residual endotoxin was determined by LAL assay (Associates of Cape Cod, Inc., Cape Cod, MA). The eluent was dialyzed in a 30 kDa cut-off dialysis bag against phosphate buffered saline (PBS), pH 7.2 containing 15 % glycerol, and stored at −80 °C. In all experiments, protein quantification was determined by BCA Protein Assay Kit (Thermo Pierce). The 104-kDa rlipoA-RBD was separated by 8 % SDS-PAGE. Samples separated in the gel were transferred onto PVDF membrane (GE). PVDF membrane was blocked with 5 % nonfat dry milk (w/v) in PBS for 1 h. To specifically identify rlipoA-RBD, the membrane was inoculated with anti-his tag (AbD Serotec; Kidlington, UK) or anti-TcdA antibodies (Clone PCG-4; GenTax, Taiwan) in PBS containing 1 % nonfat dry milk (w/v) for 1 h. After washing twice with PBST (PBS containing 0.05 % Tween 20), HRP-conjugated secondary antibodies in PBS containing 1 % milk was added and incubated for 1 h. Membrane was washed twice with PBST and developed using Luminata Crescendo substrate according to manufacturer’s instruction (Millipore, Billerica, MA). The lipid moiety of rlipoA-RBD was also analyzed using mass spectroscopy [32].

### Surface markers and cytokines analyses for DC maturation

Analysis of DC maturation was performed *in vitro* as previously described [30, 33]. C57BL/6 mice were purchased from National Animal Center in Taiwan and held at the Animal Center of the NHRI. In brief, bone marrow-derived DCs (BMDCs) were collected from the tibiae of 6 to 8-week old C57BL/6 females. Bone marrow cells were isolated by vigorous washing with LCM (RPMI 1640 containing 1 % antibiotics with penicillin and streptomycin, 10 % heat-inactivated FBS, 50 μM β-mercaptoethanol, and 50 mM HEPES) and treated with lysis buffer to remove erythrocytes. BMDC were re-suspended at 2 × 10^6^ cells per mL in LCM and treated with a final 20 ng/mL of recombinant granulocyte macrophage colony stimulating factor (MoGM-CSF) (Peprotech, Rocky Hill, NJ) on days 0 and 3. An aliquot of suspended BMDCs equivalent to 1 × 10^6^ cells/mL was seeded into 24-well plates on day 6. Different concentrations of rlipoA-RBD with or without 10 ng of polymyxin B were added into the wells. LPS (1000 EU, Sigma-Aldrich) served as the control. After 16 to 18 h incubation at 37 °C, BMDCs were analyzed by flow cytometry (FACSCalibur, BD Biosciences, Franklin Lakes, NJ) to evaluate the up-regulation of cell surface markers. In order to exclude immature DCs, which represent 50 % of the total cell population, the CD11c^+^ cell population was gated for surface marker staining with specific monoclonal antibodies to CD-40, CD-80, CD-86, and MHC-II. In addition, cell culture supernatants were collected for cytokine expression. Cytokines such as IL-6, IL-12p40 and TNF-α were determined using specific cytokine kits purchased from eBioscience (San Diego, CA). All experiments were performed at least three times. To eliminate the DC activation by rlipoA-RBD is not mouse strain specific, similar experiments were performed with BMDCs obtained from BALB/c mice.

### Antigen immunogenicity in the mouse model

BALB/c mice were purchased from the National Animal Center in Taiwan and held at the Animal Center of the NHRI. Groups of mice (6 BALB/c mice per group) were vaccinated with three intramuscular injections of various amounts of either (a) rlipoA-RBD (3, 10 or 30 μg) or (b) A-rRBD (3, 10 or 30 μg) every two weeks. Before each immunization (week 0, week 2, week 4 and week 6), mice were bled by tail vein to collect sera that were stored at −20 °C before used in anti-RBD antibody titer determination. To study the adjuvant effect of rlipoA-RBD, individual groups of 4 BALB/c mice were immunized intramuscularly with 2 μg of ovalbumin (OVA) (Sigma-Aldrich) formulated either with various amounts of rlipoA-RBD (0.3, or 3 μg), or 10 μg of A-rRBD or alum (Sigma-Aldrich). Animals that received 2 μg of OVA alone served as the control. The mice were given three immunizations at two week intervals and bled before each injection. Sera were collected and stored at −20 °C for anti-OVA antibody titer measurement using OVA-specific ELISA as described below.

### Rabbit immunogenicity study

New Zealand white (NZW) rabbits with 1.6–to 2.0- kg body weight were purchased from Livestock Research Institute in Taiwan and held at the Animal Center of the NHRI for experiments. Groups of two NZW rabbits were intramuscularly vaccinated with 10 μg of either rlipoA-RBD or A-rRBD formulated with alum three times, 14 days apart. Before each immunization (week 0, week 2, week 4 and week 6), rabbits were bled via the central ear artery. Sera were collected and stored at −20 °C for further analyses.

### Antigen-specific ELISA

ELISA plate wells were coated either with 100 ng of A-rRBD or OVA at 4 °C overnight, then blocked with 5 % nonfat dry milk (w/v) in PBS. Mouse antisera 2-fold serially diluted with PBS containing 1 % BSA (Calbiochem, Darmstadt, Germany) were added to the wells followed by incubation at room temperature (RT) for 2 h. After washing with 3 × PBST, either anti-IgG isotypes (Invitrogen, Carlsbad, CA.) or anti-IgA (Invitrogen, Carlsbad, CA) HRP-conjugated IgG (KPL, Gaithersburg, MD) specific antibodies diluted in PBS containing 1 % BSA were added to the wells and incubated at RT for 1 h. After washing with 3 × PBST, the plates were treated with TMB peroxidase substrate (KPL) at room temperature in the dark for 20 min. To determine anti-A-rRBD or anti-OVA titer, OD_450nm_ absorbance was measured using a spectrophotometer (Spectra max M2, Molecular Devices, Sunnyvale, CA).

### Anti-toxin neutralization assay

The anti-TcdA neutralization assay was performed according to the protocol previously described by Huang et al. [31]. Briefly, Vero cells (2 × 10^4^ per well) were seeded into 96-well plates containing VP-SFM culture medium (Invitrogen, Carlsbad, CA) and 4 mM glutamine at 37 °C, and allowed to grow to confluent. Mouse sera from mice immunized either with rlipoA-RBD or A-rRBD or B-rRBD were serially diluted two-fold with fresh VP-SFM and mixed with an equal volume of either 20 ng/mL TcdA or 40 pg/mL of TcdB (The Native Antigen Company Ltd, Oxfordshire, UK) and incubated at room temperature for 1 h. The mixture was added to the 96-well plates containing Vero cells and incubated at 37 °C for 24 h. Anti-TcdA neutralization titers were calculated as the highest serum dilution which could protect 50 % of cells from rounding due to toxin cytotoxicity. Cellular toxicity was recorded using a microscope equipped with a camera.

### TcdA challenge in the mouse model

A lethal TcdA challenge mouse model was established to assess the efficacy of anti-RBD immune responses *in vivo* using the protocol previously described by Seregin et al. [21]. Briefly, groups of BALB/c mice (10 mice per group) were immunized intramuscularly with either PBS or three dosages of various amounts of immunogens (rlipoA-RBD (0.3 or 3 μg), A-rRBD (0.3 or 3 μg) or B-rRBD (30 μg)) without adjuvant at days 0, 14, and 28. After three immunizations, mice were challenged with 150 ng of TcdA (5 × Lethal Dose (LD_50_)), by intra-peritoneal injection on day 35 and monitored for 14 days. The mice were observed twice daily during the first 3 days.

### Preparation of *C. difficile* spores and hamster challenge model

The protocol for preparation of *C. difficile* spores was modified from the method previously reported by Lyras et al., [34]. Briefly, *C. difficile* strains VPI10463 were streaked on 10 anaerobic blood agar plates and grown anaerobically at 37 °C to induce sporulation at around the 5th or 6th day. The cells were harvested with disposable loops and washed in 10 mL PBS, and heat-shocked at 56 °C for 30 min to kill surviving vegetative cells. The spores were collected by low-speed centrifugation and resuspended in DMEM, aliquoted and frozen at −80 °C. The frozen spores were then quantified before use by plating ten-fold serial dilutions of the spores onto Taurocholatefructose-agar (TFA) plates which were prepared with agar plus taurocholate-cefoxitin- cycloserinefructose-agar (TCCFA) without cycloserine and cefoxitin. Hamster challenge model was performed as follows. Six hamsters per group (6 weeks old and weighed 100–130 g) were purchased from National Animal Center in Taiwan and held at the Animal Center of the NHRI. Groups of hamsters were vaccinated with three intramuscular injections of either (a) rlipoA-RBD (10 μg) alone, (b) A-rRBD (10 μg) alone, (c) B-rRBD (10 μg) alone, (d) rlipoA-RBD (10 μg) + B-rRBD (10 μg), A-rRBD (10 μg) + B-rRBD (10 μg) formulated with (e) 300 μg of aluminum phosphate (alum) or (f) 10 μg of Pam3CSK4 (InvivoGen, San Diego, CA) every two weeks. Before each immunization, hamster blood sera were carefully collected by the heart puncture and stored at −20 °C before used in anti-RBD antibody titer determination. After three immunizations as described above, hamsters were given clindamycin orogastrically (30 mg/kg) to render them susceptible to *C. difficile* infection (day 0). On day-5 post clindamycin treatment, hamsters in each group were gastrically inoculated with 100 cell forming unit (CFU) of *C. difficile* spores, and monitored twice daily for 5 days and then daily thereafter. Animal bedding was changed and faecal pellets were collected every two days. Specimens were inoculated onto selective TCCFA plates and incubated anaerobically at 37 °C to determine if they were colonized with *C. difficile*. Faecal pellets were collected every two days for 12 days, then weekly until the study terminated (at least 14 days). Each hamster group was assessed for *C. difficile* colonization and survival rate.

### Statistical analysis

Data was expressed using Prism 5 version 5.01 (GraphPad Software, Inc.). Antibody titer was displayed as means ± SEM from the experiments. Statistical difference was analyzed using a two-tailed students’ *t* test by comparison of the means obtained in each treatment with the control group.

## Results and discussion

### Production of rlipoA-RBD

**Fig. 1:**
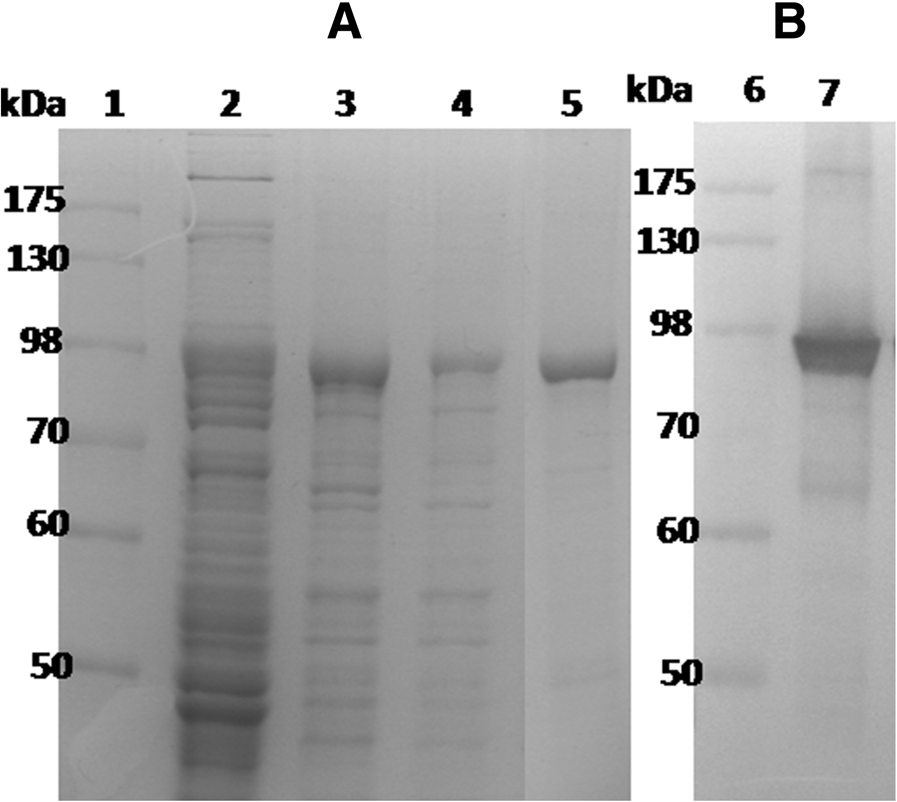
The expression and purification of rlipoA-RBD. The expression and the purity of rlipoA-RBD were confirmed by SDS-PAGE (**a**) and western blot with TcdA-specific monoclonal antibody (**b**). The purification of rlipoA-RBD as shown in panel **a**, lanes 2 to 4 were crude extract loaded on the first Ni-affinity column, eluent of 200 and 500 mM imidazole, respectively. The purity of rlipoA-RBD obtained from IMAC were confirmed by SDS-PAGE (panel **a**, lane 5) and western blot with TcdA-specific monoclonal antibody (panel **b**, lane 7). The first lane in each panel was the molecular markers

We have previously reported that both A-rRBD and B-rRBD at 0.8 −1 μM have strong abilities to up-regulate cell surface marker expression and cytokine secretion from BMDCs [30, 31]. To enhance the effectiveness of A-rRBD as a recombinant subunit vaccine candidate against CDI, A-rRBD was rationally designed and lipidated (rlipoA-RBD) to contain a toll-like receptor 2 agonist (intrinsic adjuvant) [32]. Construction of plasmid containing rlipoA-RBD was cloned into the pET-22b(+) vector as described in the Materials and Methods, and the construct was successfully expressed in *E. coli* C43 (DE3) strain. After the first single-step purification using Ni-affinity chromatography, purified rlipoA-RBD with expected molecular weight closed 100 kDa (>85 % purity) was obtained and its purity confirmed by SDS-PAGE (Fig. 1a, lanes 3 & 4). Most of the *E. coli* proteins and endotoxin (LPS) were successfully removed by binding the rlipoA-RBD preparation to the second IMAC-affinity column and washing with PBS containing 0.1 % Triton-X100. The purity of eluted rlipoA-RBD was confirmed by SDS-PAGE (Fig. 1a, lane 5) and the western blot analysis using a TcdA-specific monoclonal antibody (Fig. 1b, lane7). Trace amounts of rlipoA-RBD degradation fragments were also detected using TcdA-specific antibody (Fig. 1b). These degradation products are likely the result of proteolytic digestion during the purification process. The residual LPS in the purified rlipoA-RBD were found to be below 30 EU per mL based on the Limulus assay. In any event, at least 5−10 mg of highly purified rlipoA-RBD was easily obtained from 1 l of bacterial culture.

**Fig. 2:**
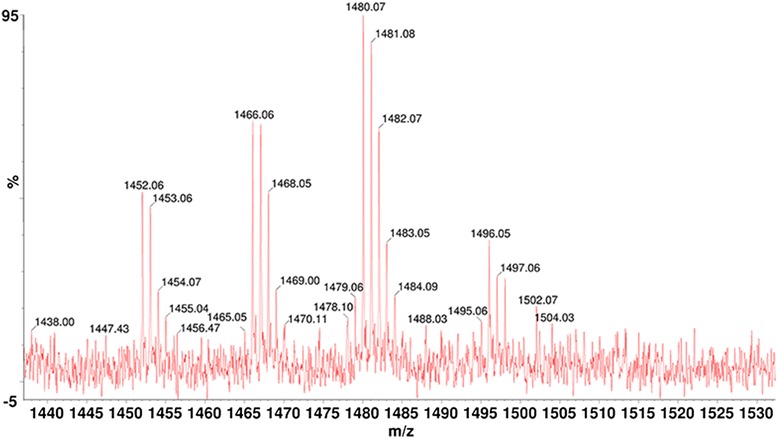
MALDI-TOF analysis of tryptic digested lipopeptide fragments of rlipoA-RBD. The N-ternminal tryptic digested fragments were analyzed by MLADI-TOF using the procedure described in previous report [32]

The lipid moiety of rlipoA-RBD was identified using mass spectroscopy analysis [32]. The purified rlipoA-RBD was digested with trypsin and the tryptic fragments were analyzed using MALDI-TOF. Typical groups of ion mass peaks which exhibit the post-translational modification signature of recombinant lipoprotein, contain three peaks with m/z values of 1452, 1466, and 1480 as shown in Fig. 2. The mass differences between these peaks are 14 amu and the pattern of isotopes in each group is exactly identical to that previous report [32]. The circular dichroism (CD) secondary structure analysis of rlipoA-RBD was also performed and found that rlipoA-RBD had correctly folded to form β-sheet structure similar to A-rRBD (>43 %) [30]. This result is consistent with other reports that RBD forms stable folded β-solenoid secondary structures independently of other functional domains in the TcdA [30, 31]. Although a simple and rapid method for producing rlipoA-RBD with high purity was successfully developed, rlipoA-RBD was found to be unstable and showed a loss in biological function during the freeze-thaw process. The best condition for preserving rlipoA-RBD integrity was to store the protein at 1 mg/mL in PBS containing 10 % (v/v) of glycerol at −80 °C.

### Dendritic cell maturation triggered by rlipoA-RBD

**Fig. 3:**
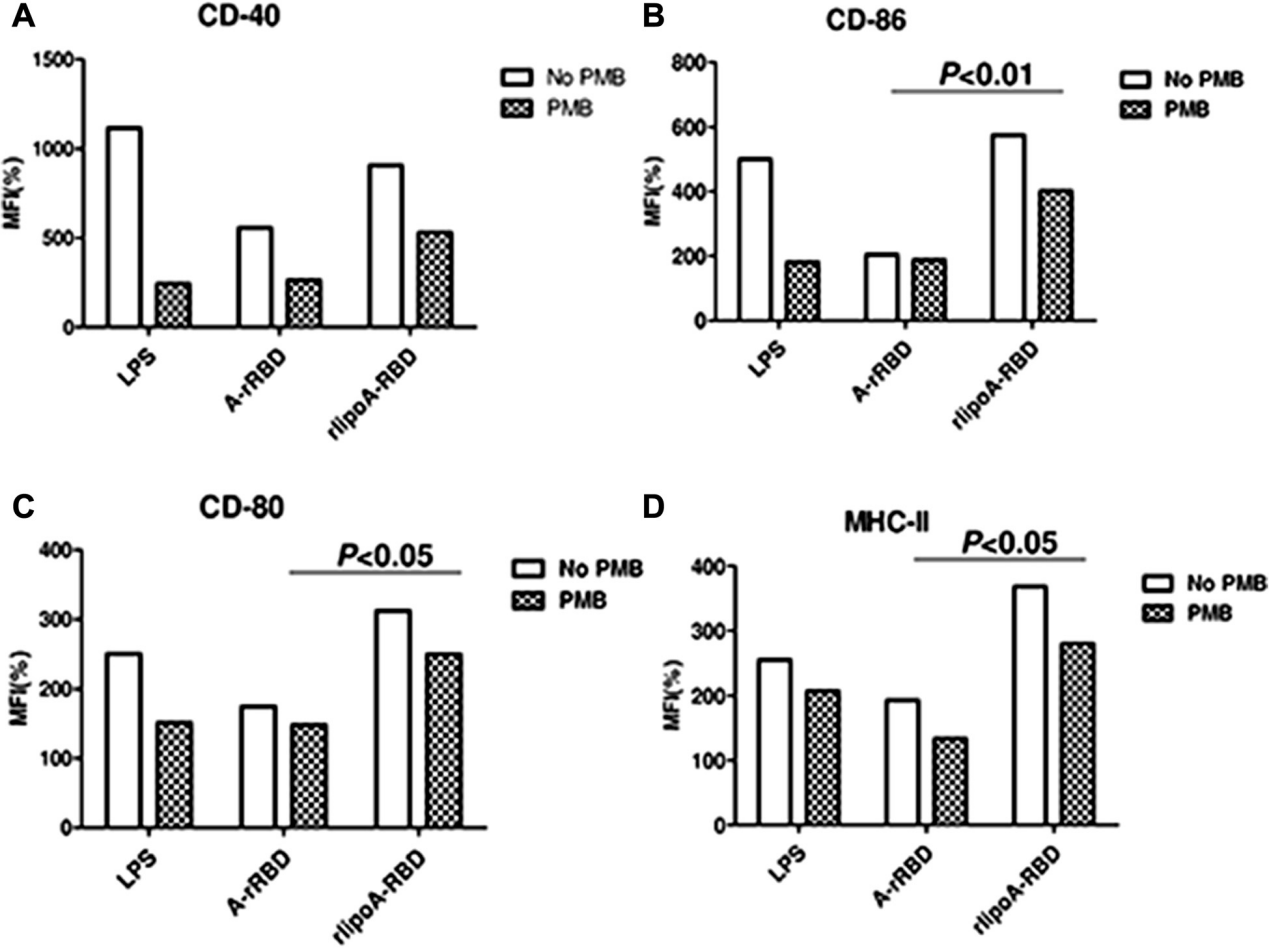
Up-regulation of surface biomarkers of BMDC by rlipoA-RBD. BMDC from C57BL/6 was collected and treated with GM-CSF on days 0 and 3. A-rRBD and rlipoA-RBD were treated on day 6 for 18 h, then DC were collected to analyze their surface markers, including CD-40 (**a**), CD-86 (**b**), CD-80 (**c**), and MHC-II (**d**) by flow cytometry. All groups were divided into polymyxin B (PMB) treated (black-net bar) or without (white bar) to validate insignificant LPS contamination. All surface marker signaling was normalized by calculating the ratio of mean fluorescence intensity (MFI) between medium control and treatments. The experiments had been performed at least three times

**Fig. 4:**
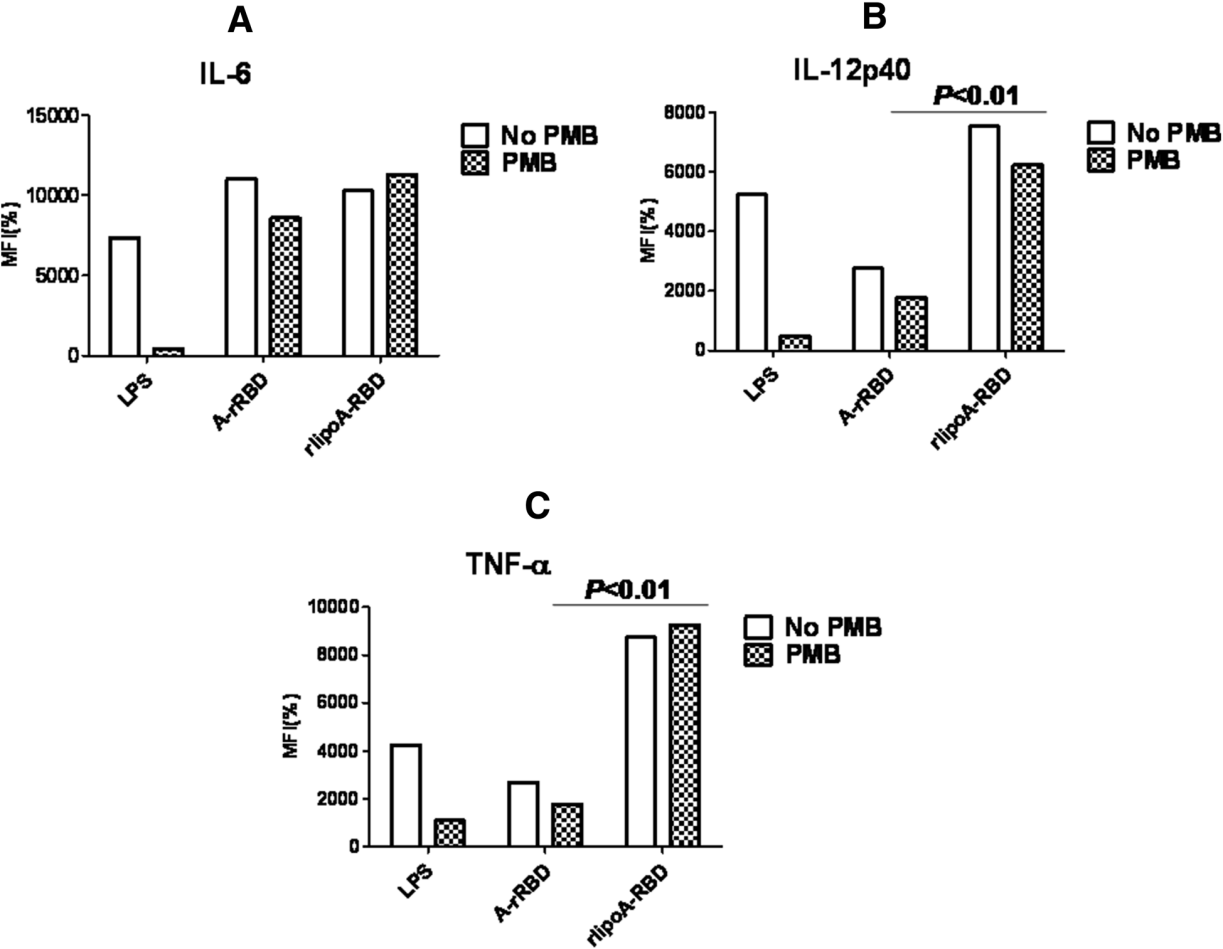
Cytokine secretion from BMDC treated with rlipoA-RBD. After BMDC was treated with rlipoA-RBD on day 6 for 18 h, the culture supernatants were collected and analyzed for cytokine profiles using specific cytokine ELISA: (**a**) IL-6, (**b**) IL12p40, and (**c**) TNF- α. The experiments had been performed at least three times

rlipoA-RBD was tested for its ability to promote the maturation of DC. BMDCs from C57BL/6 mice were treated with increasing amounts (0.2 to 2 μM) of rlipoA-RBD, cell surface biomarkers associated with DC maturation (CD40, CD80, CD86, and MHC-II) and the secretion of pro-inflammatory cytokines (IL-6, IL-12, and TNF-α) were examined using FACS analysis and cytokine-specific ELISA, respectively. In order to preclude the interference of LPS contamination, even though rlipoA-RBD samples used in the current studies had very little LPS contamination (0.03 EU/μg of protein), polymyxin B was added to DC samples to prevent activation by LPS through the Toll-like receptor 4 pathways. It was found that surface biomarkers of DC maturation were up-regulated and that the production of pro-inflammatory cytokines (IL-6, IL-12, and TNF-α) increased in a dose-dependent manner (data not shown). A 0.5 μM of rlipoA-RBD in the final assay solution was selected to perform subsequent experiments and compare with the DC activation obtained from 0.5 μM of A-rRBD. Both the biomarker up-regulation (CD80, CD86 and MHC II) of DC maturation and the production of pro-inflammatory cytokines (IL-12, and TNF-α) were detected significantly higher in the rlipoA-RBD-treated BMDCs than those obtained with A-rRBD (Fig. 3 & Fig. 4). No difference is repeatedly observed for IL-6 that is a surprise. The current results nevertheless strongly indicate that the intrinsic adjuvant properties of rlipoA-RBD are significantly (p < 0.05) more potent than A-rRBD. As observed DC biomarker activation in Fig. 3, there are differences between polymyxin B treated and non-treated sample but these differences are not significant. In contrast, the results in the production of the pro-inflammatory cytokines were not influenced by minor LPS contamination as there was no significant difference between polymyxin B treated and non-treated samples as shown in Fig. 4. However, when both rlipoA-RBD samples and LPS were boiled and tested for their biological functions, boiling did not affect LPS-induced DC activation but fully abolished rlipoA-RBD DC-activation ability (data not shown). Overall, this clearly demonstrates that DC activation is mediated by rlipoA-RBD. To eliminate the DC activation by rlipoA-RBD is not mouse strain specific, similar results were obtained when BMDCs from BALB/c mice were examined for cell surface biomarkers associated with DC maturation and the secretion of pro-inflammatory cytokines using FACS analysis and cytokine-specific ELISA (data not shown). The results are consistent with our previous reports [30–32].

### Adjuvant property of rlipoA-RBD

**Fig. 5:**
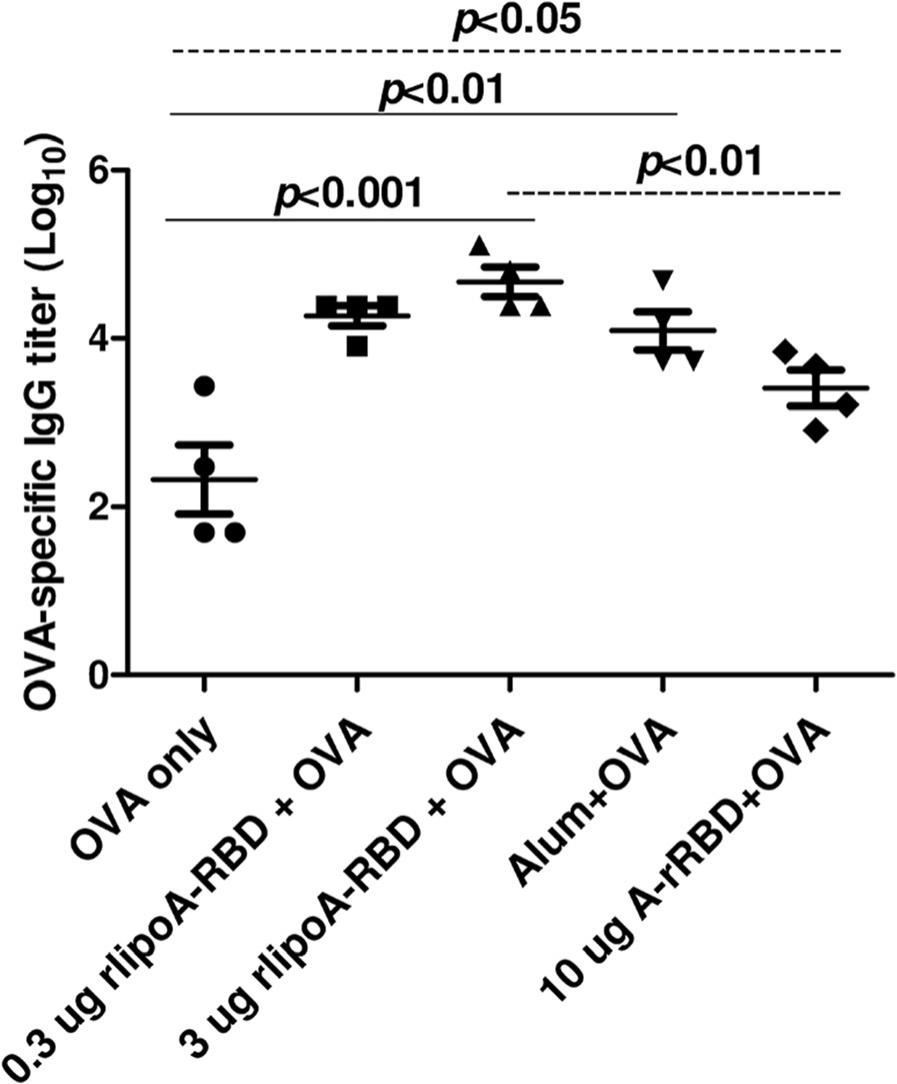
Adjuvant effect of rlipoA-RBD. To demonstrate adjuvant effect of rlipoA-RBD, the enhancement of anti-OVA IgG response was evaluated by co-administration of rlipoA-RBD and OVA. BALB/c mice were immunized with 3 × 2 μg of OVA formulated either with or without various amounts of rlipoA-RBD (0.3 and 3 μg), 10 μg of A-rRBD, or alum as positive control. Serum titer was determined by RBD-specific ELISA

To investigate whether rlipoA-RBD could provide an adjuvant effect and enhance immune responses against poor immunogens such as ovalbumin (OVA), mouse immunogenicity studies were performed using OVA (2 μg) formulated with rlipoA-RBD (3 μg). A ten-fold increase in mouse anti-OVA IgG titers (>10^4^) was observed by formulating OVA with rlipoA-RBD as compared to those obtained with OVA alone (Fig. 5). To further investigate the adjuvant activity of rlipoA-RBD, mouse immunogenicity studies were repeated with OVA formulated with either 0.3 or 3 μg of rlipoA-RBD, 10 μg of A-rRBD, or alum (300 μg). Interestingly, even a dose as low as 0.3 μg of rlipoA-RBD exhibited a 10-fold increase in OVA-specific IgG titers over those obtained with OVA alone (Fig. 5). The increase in anti-OVA responses was shown to be statistically significant (*p* < 0.001). There was no significant difference in the immunogenicity of OVA formulated in rlipoA-RBD or alum, but a significant difference was observed in the anti-OVA IgG antibody responses elicited by rlipoA-RBD and A-rRBD *(p* < 0.01) (Fig. 5). Again, these results indicate that rlipoA-RBD provides stronger adjuvant activities and enhancing immune responses against weak immunogens compared to A-rRBD.

### Mouse immunogenicity of rlipoA-RBD

**Fig. 6:**
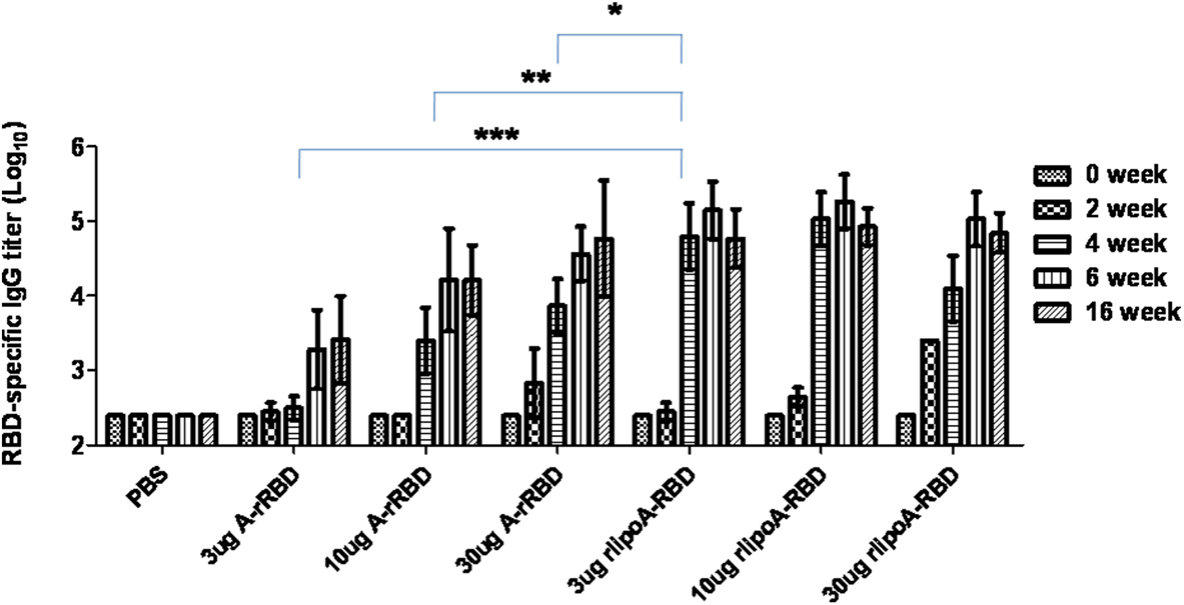
Mouse anti-RBD antibody responses elicited by different dosages of A-rRBD or rlipoA-RBD. BALB/c mice were immunized three times with either 3, 10 or 30 μg doses of A-rRBD; or with 3, 10 or 30 μg doses of rlipoA-RBD. Anti-RBD titers of mouse sera obtained at 0, 2, 4, 6, and 16 weeks were determined by RBD-specific ELISA

Our previous studies [30, 31] had indicated that A-rRBD (10 μg) and B-rRBD (10 μg) alone without adjuvant could elicit toxin-specific neutralizing antibody responses in both mouse and rabbit models. To assess the immunogenicity of rlipoA-RBD, groups of mice (6 BALB/c mice per group) were vaccinated with various amounts of rlipoA-RBD. Analyses of mouse antisera from each immunization using RBD-specific ELISA revealed that three doses of 3 μg of rlipoA-RBD already induced very strong anti-RBD IgG antibody response (Fig. 6). The results shown in Fig. 6 also indicate that rlipoA-RBD is more immunogenic than A-rRBD as rlipoA-RBD (2 x 3 μg) elicited stronger anti-rRBD IgG antibodies (titer >10^5^ at Week 4) than those immunized with 2 x 30 μg of A-rRBD (*p* < 0.01). Moreover, antisera from mice vaccinated with 3 μg of rlipoA-RBD both IgG_1_ and IgG_2_ isotypes antibody responses were observed (data not shown). Post 6 week (after 3 doses) vaccination, anti-A-rRBD IgG antibody titers (~3 × 10^5^) elicited by 3 μg of rlipoA-RBD were not different from those obtained with either 3 × 30 μg of A-rRBD or 3 × 10 μg of TcdA toxoid (Table 1). Mouse antisera obtained from mice vaccinated with 2 doses of 3 μg of rlipoA-RBD was capable of inducing >10^5^ anti-RBD IgG titer (Fig. 6) which strongly supports rlipoA-RBD as a good vaccine candidate.

**Table 1:** Antibody responses of antisera obtained two weeks after 3 doses of different formulation of rlipoA-RBD in the mouse, rabbit and hamster immunogenicity studies

Immunogenicity studies			Anti-A-rRBDIgG titer of pooled sera	Neutralization titer of pooled sera against^a^	
				TcdA	TcdB
Mouse	A-rRBD (μg)	0	<100	<4	<4
		3	3.5 × 10^3^	32	<4
		10	2.7 × 10^4^	256	<4
		30	5.3 × 10^4^	128	<4
	rlipoA-RBD (μg)	0.3	2.3 × 10^5^	512	<4
		3	2.3 × 10^5^	512	<4
		10	3.3 × 10^5^	512	4
		30	2.5 × 10^5^	1024	4
	TcdA toxoid^b^ (μg)	10	7.7 × 10^4^	256	<4
Rabbit	A-rRBD (μg)	10	2.3 × 10^5^	128	<4
	rlipoA-RBD (μg)	10	7.7 × 10^5^	512	<4
	TcdA toxoid^b^ (μg)	10	7.7 × 10^4^	2048	<4
Hamster	A-rRBD (μg)	10	3.7 × 10^4^	128	<4
	B-rRBD (μg)	10	2 × 10^3^	8	64
	rlipoA-RBD (μg)	10	5.7 × 10^5^	512	8
	A-rRBD (μg) + B-rRBD^c^ (μg)	10	2.7 × 10^4^	32	16
	rlipoA-RBD (μg) + B-rRBD (μg)	10	7.7 × 10^5^	512	64
	A-rRBD (μg) + B-rRBD (μg) + alum^c^	10	4.3 × 10^5^	64	64
	A-rRBD (μg) + B-rRBD (μg) + Pam3CSK4^d^	10	1.7 × 10^5^	32	8

^a^Neutralization titer of pooled sera against toxins was defined as the highest diluted sample which could protect against 50 % cell rounding induced by toxins

^b^Mouse antibody responses to 3 doses of 10 μg of TcdA toxoid. The immunogenicity studies were performed and described in previous report [30]

^c^Hamster antibody responses to 3 doses of 10 μg of each A-rRBD (μg) and B-rRBD formulated with 300 μg of alum

^d^Hamster antibody responses to 3 doses of 10 μg of each A-rRBD (μg) and B-rRBD formulated with 10 μg of Pam3CSK4

### Rabbit immunogenicity of rlipoA-RBD

To avoid animal-specific immune responses and further assess the immunological properties of rlipoA-RBD, rabbit immunogenicity was performed. Groups of 2 rabbits were vaccinated either with 10 μg of A-rRBD, rlipoA-RBD or TcdA toxoid. The results indicated that rlipoA-RBD is also highly immunogenic in rabbits since 2 × 10 μg (2 doses) of rlipoA-RBD alone produced anti-A-rRBD IgG antibodies with an average titer >10^5^, that was comparable to antisera from rabbits vaccinated 3 times with A-rRBD (data not shown). Rabbit anti-rRBD IgG antibody responses elicited by 3 × 10 μg of rlipoA-RBD were higher than those obtained with either 3 × 10 μg of A-rRBD alone or 3 × 10 μg of TcdA toxoid (Table 1). These results clearly indicate that 10 μg of rlipoA-RBD is enough to induce strong anti-rRBD IgG antibody responses in rabbits.

### Functional roles of anti-A-rRBD sera elicited by rlipoA-RBD

To determine whether mouse and rabbit anti-A-rRBD antibodies elicited by rlipoA-RBD could functionally neutralize the cytotoxicity of *C. difficile* TcdA and TcdB, both mouse and rabbit antisera were tested in a Vero cell cytotoxicity assay as described in Materials and Methods. As shown in Table 1, antisera from both mice and rabbits immunized with 3 x 10 μg of rlipoA-RBD were capable of preventing 50 % of cell death resulting from TcdA cytotoxicity at 1/512 dilution. This was not significantly better than the results obtained from antisera of animals immunized with 3 x 10 μg of A-rRBD (1/256). However, the neutralization titers (1/512) obtained from mice immunized 3 × 3 μg of rlipoA-RBD were found to be significantly higher (p < 0.01) as compared to those obtained from 3 × 3 μg of A-rRBD alone (1/32) (Table 1). Anti-toxin neutralization titer obtained from mouse sera with 30 μg of rlipoA-RBD was significantly higher (p < 0.01) than those elicited either by 30 μg of A-rRBD alone or 10 μg of TcdA toxoid (Table 1). Nevertheless, the current results indicate that 3 μg of rlipoA-RBD alone was enough to induce significant functional neutralizing antibody levels against TcdA. Interestingly, these anti-RBD IgG antibody responses had little or no neutralization activity against TcdB (Table 1). Also, the anti-RBD IgG antibody responses elicited by freeze-thaw or heat-treated rlipoA-RBD were found to be significant lower or have no neutralizing activity (data not shown). Again, preserving the functionally active conformation of rlipoA-RBD is vital to achieving neutralization activity.

**Fig. 7:**
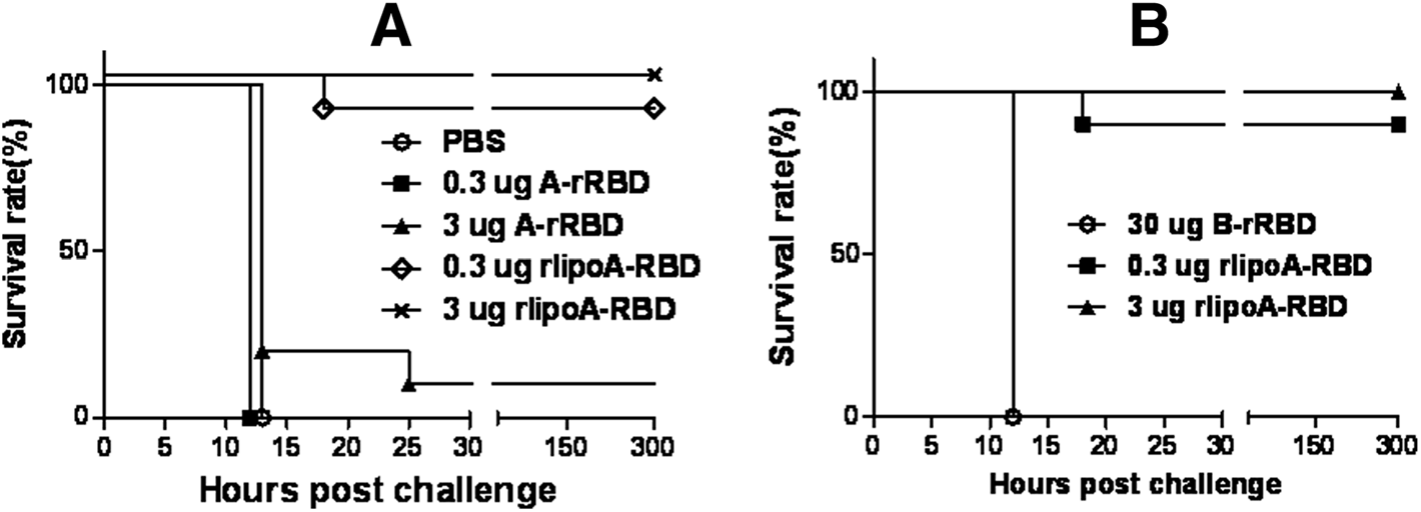
Mouse protection elicited by rlipoA-RBD against lethal TcdA challenge. Panel **a**, BALB/c mice (10 mice per group) were challenged with a lethal dose of TcdA after three immunizations of either rlipoA-RBD or A-rRBD (0.3 and 3 ug). PBS served as the negative control. The final survival rates were reported. Panel **b**, BALB/c mice (10 mice per group) were challenged with a lethal dose of TcdA after three immunizations of either rlipoA-RBD (0.3 and 3 μg) or 30 μg of B-rRBD

To further evaluate the role of this anti-toxin neutralizing activity *in vivo*, mice were immunized 3 times with increasing doses of rlipoA-RBD (0.3, 3, or 30 μg) and challenged with 5 times the dose killing half of the subjects (LD_50_) of TcdA. Low dose vaccination (0.3 μg) induced a strong anti-RBD antibody response which could neutralize TcdA *in vitro* Vero cell cytotoxicity assay (neutralization titer 1/128) and fully protected immunized mice against TcdA challenge. Our previous study [30] indicated that 3 × 10 μg of A-rRBD was capable of a >80 % protection rate in the TcdA mouse challenge model, so to determine whether lower doses could provide protection the challenge studies were repeated with groups of mice (10 mice per group) vaccinated with either 3 × 0.3 or 3 × 3 μg of either A-rRBD or rlipoA-RBD. The protection rates obtained with 0.3 and 3 μg of A-rRBD were found to be 0 and 10 %, respectively (Fig. 7a). In contrast, the protection rates obtained with 0.3 and 3 μg of rlipoA-RBD were 90 and 100 %, respectively (Fig. 7a). In another separate experiment, mice (10 mice per group) were vaccinated with 3 × 0.3 or 3 × 3 μg of rlipoA-RBD, or with 3 × 30 μg of B-rRBD, the protection rate was found to be 90 %, 100 % and 0 % for 3 × 0.3 rlipoA-RBD, 3 × 3 μg and B-rRBD groups, respectively (Fig. 7b). A low neutralizing antibody titer (1/16) against TcdA was detected in mouse sera elicited by 3 × 30 μg of B-rRBD, and these antibodies were insufficient to protect mice from TcdA challenge *in vivo*. Taken together, the results demonstrate that 3 × 0.3 μg of rlipoA-RBD elicits neutralization titer >128 and provides full protective immune responses in mice against *C. difficile* TcdA challenge, and strongly suggests rlipoA-RBD is a good candidate for CDI vaccine developments.

### Hamster challenge studies

To further evaluate the roles of anti-toxin neutralizing antibodies *in vivo*, the *C. difficile* spore hamster challenge model was performed as described in the Materials and Methods. Two groups of hamsters (*n* = 6) were vaccinated 3 times with PBS 2 weeks apart (one group is used for challenge as the positive control and one group has no challenge as the negative control) and another three separated groups of hamsters were immunized with either 10 μg of A-rRBD, or 10 μg of rlipoA-RBD or 10 μg of B-rRBD intramuscularly. A week after the third immunization, blood samples collected from immunized hamster were assayed for anti-TcdA neutralizing antibdoy titers and found to be <4, 8, 128 and 512 for PBS, B-rRBD, A-rRBD and rlipoA-RBD groups, respectively (Table 1). As shown in Table 1 only hamster antisera obtained from the group immunized with B-rRBD had strong anti-TcdB neutralizing antibody response (titer = 64). The hamster anti-TcdA titers elicited by rlipoA-RBD were comparable to those obtained from mice and rabbits. Two weeks after the third immunization, hamsters were gastrically inoculated with 100 CFU (the dose can kill >50 % of challenged hamsters) of *C. difficile*. After 3 to 4 days 6/6, 6/6, 5/6 and 5/6 of hamsters died in the PBS, A-rRBD, rlipoA-RBD and B-rRBD groups, respectively (data not shown). These results indicate that A-rRBD and B-rRBD alone could not elicit protective immune responses in hamster challenge model and are consistent with those results obtained from previous studies [22, 31].

**Fig. 8:**
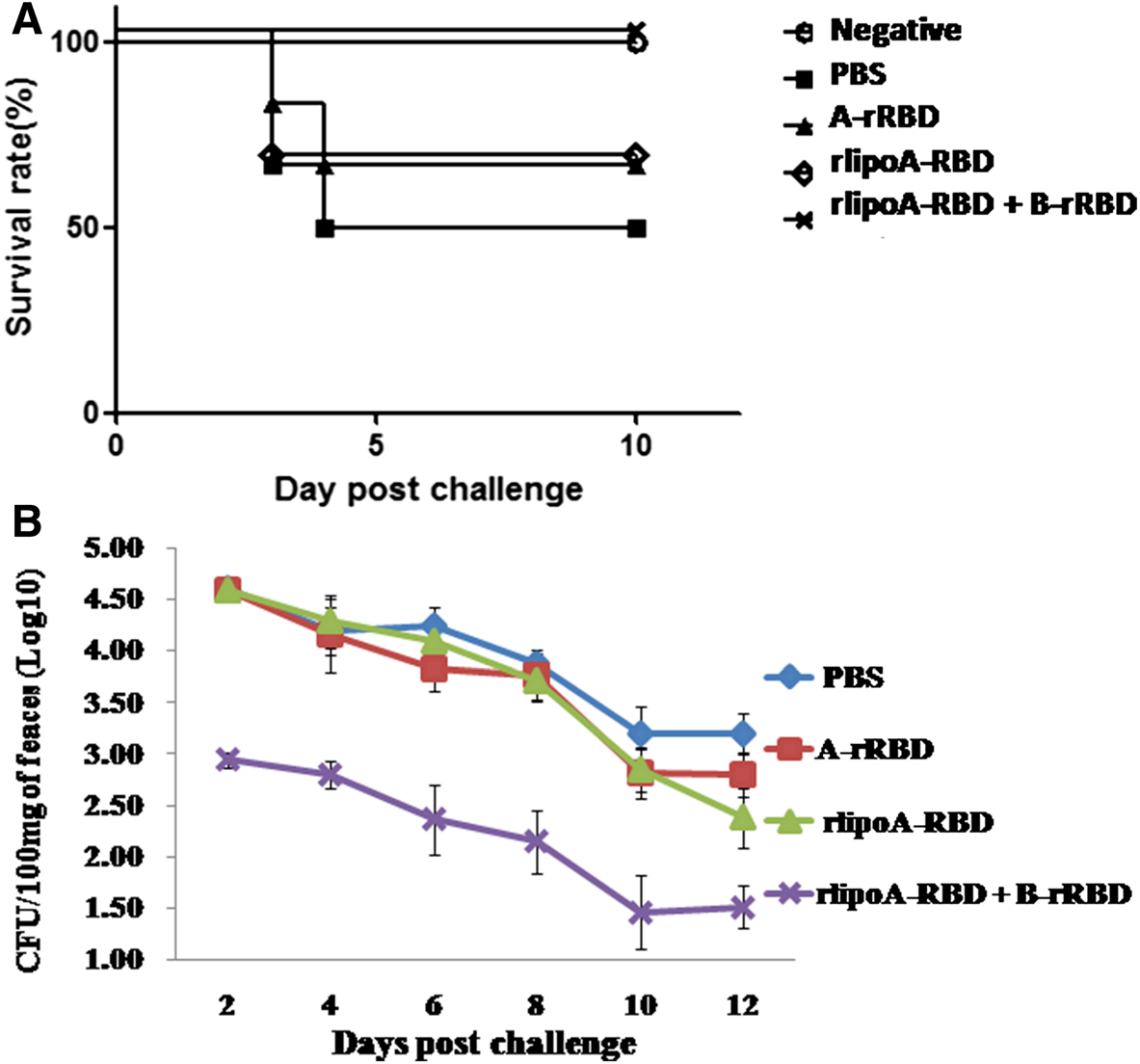
*C. difficile* spore challenge in hamster model studies. Panel **a**, five groups of hamsters (n = 6) were gastrically inoculated with 100 CFU of *C. difficile* (the dose can kill >50 % of hamsters) at 2 weeks after the third immunization with either PBS, A-rRBD, rlipoA-RBD, or rlipoA-RBD + B-rRBD. The final survival rates were reported. Panel **b**, The number of *C. difficile* colonies (CFU) grown in TCCFA selective medium. Bacterial colonies obtained from the faeces (~100 mg) of survived hamsters were determined every two days after challenge. The number of CFU shown in the figure represented the geometric mean of CFU per 100 mg of feacal pellets obtained from survived hamsters in each group after the spore challenge

To test whether rlipoA-RBD formulated with B-rRBD could provide protective immune responses in hamster challenge model, groups of hamsters were immunized with either PBS, 10 μg of A-rRBD, or 10 μg of rlipoA-RBD with and without 10 μg of B-rRBD intramuscularly. A week after the third immunization, blood samples collected from immunized hamster were assayed for anti-TcdA neutralizing antibdoy titers and found to be <4, 128 and 512 for PBS, A-rRBD and rlipoA-RBD + B-rRBD groups, respectively (Table 1). As shown in Table 1 only hamster antisera obtained from the group immunized with rlipoA-RBD + B-rRBD had significant (*p* < 0.01) anti-TcdB neutralizing antibody (titer = 64). Two weeks after the third immunization, hamsters were gastrically inoculated with 100 CFU of *C. difficile* (dose can kill >50 % of challenged hamsters). After 3 to 4 days 3/6, 2/6, 2/6 and 0/6 of hamsters died in the PBS, A-rRBD, rlipoA-RBD and rlipoA-RBD + B-rRBD groups, respectively (Fig. 8a). Around 30 CFU of *C. difficile* were observed on selective TCCFA plates when 100 mg of the faecal pellets collected at day 12 from the six surviving hamsters immunized with rlipoA-RBD + B-rRBD were analyzed (Fig. 8b). In contrast, significant amount (500 to 7,500 CFU of *C. difficile* (p < 0.01) were found in the selective TCCFA plates when the faecal pellets (100 mg) collected from other survived hamster groups (Fig. 8b). It is clear that recombinant RBD derived from either TcdA or TcdB individually was incapable of providing total protection in the hamster challenge model and these results are consistent with previous reports [13, 17, 22, 31]. The current results indicate that rlipoA-RBD formulated with B-rRBD could provide protection that is similar to previous report that a fusion protein containing A-rRBD/B-rRBD formulated in alum adjuvant could elicit protective immune responses in hamster challenge model [22].

**Fig. 9:**
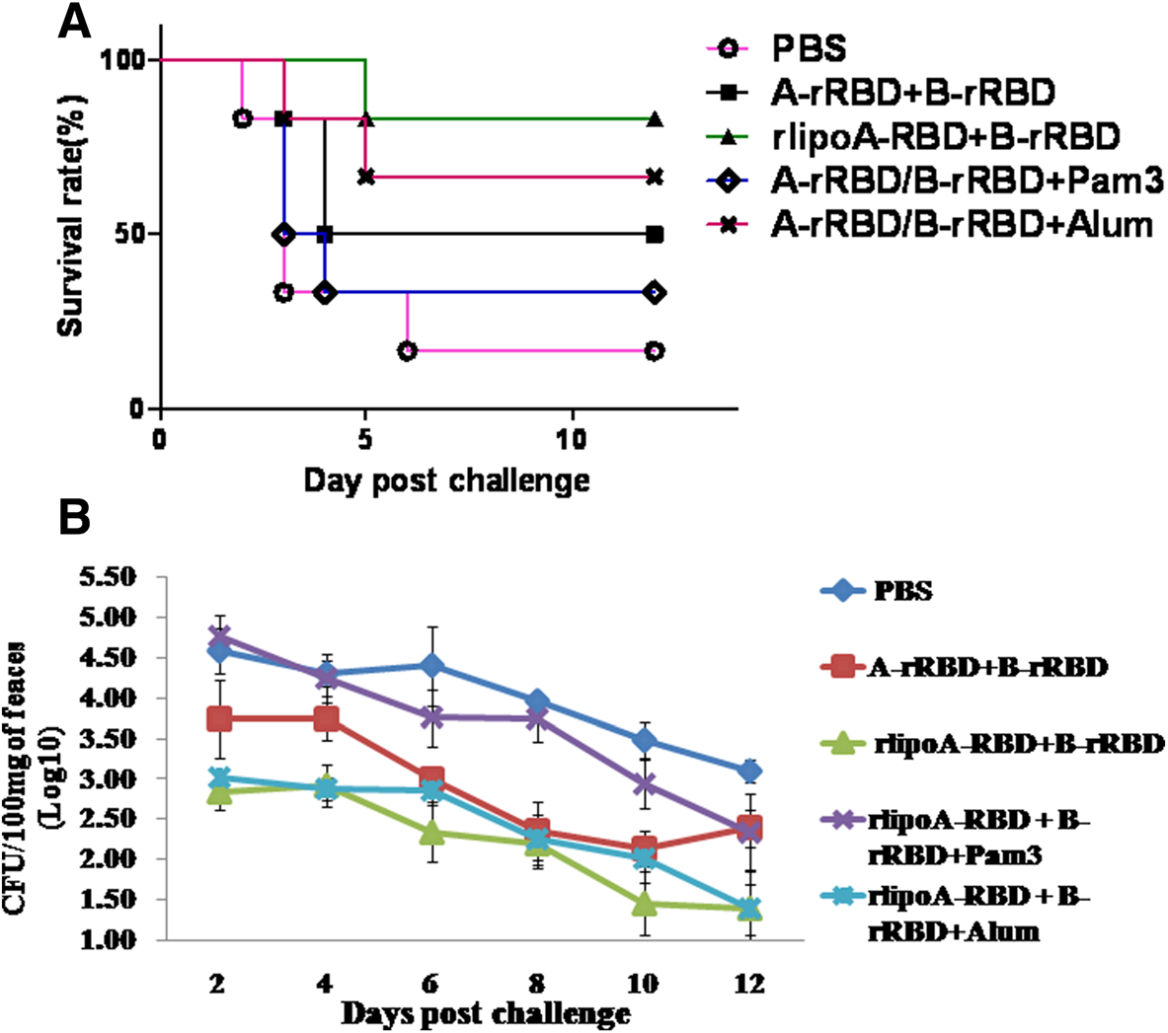
Adjuvant effect in hamster challenge model studies. Panel **a**, five groups of hamsters (*n* = 6) were gastrically inoculated with 100 CFU. of *C. difficile* (the dose can kill >50 % of hamsters) at 2 weeks after the third immunization with either PBS, A-rRBD + B-rRBD, rlipoA-RBD + B-rRBD, or A-rRBD + B-rRBD formulated with alum (300 μg) or Pam3CSK4 (10 μg). The final survival rates were reported. Panel **b**, The number of *C. difficile* colonies (CFU) grown in TCCFA selective medium. Bacterial colonies obtained from the faeces (~100 mg) of survived hamsters were determined every two days after challenge. The number of CFU shown in the figure represented the geometric mean of CFU per 100 mg of feacal pellets obtained from survived hamsters in each group after the spore challenge

To test whether adjuvant would have an impact, the hamster *C. difficile* spore challenge model was repeated with groups of hamsters vaccinated 3 times either with either PBS; 10 μg of rlipoA-RBD + 10 μg of B-rRBD; or 10 μg of A-rRBD + 10 μg of B-rRBD alone, or formulated with either alum or Pam3CSK4. Pam3CSK4 is a synthetic lipopeptide and is well recognized as a toll-like receptor 2 agnoist [32]. Two weeks after the third immunization, hamsters were gastrically inoculated with 100 CFU (the dose can kill >50 % of challenged hamsters) of *C. difficile*. As shown in Fig. 9a, the survival rate was found to be 1/6, 5/6, 3/6, 4/6 and 2/6 for PBS, rlipoA-RBD + B-rRBD, A-rRBD + B-rRBD alone, and A-rRBD + B-rRBD formulated with alum and Pam3CSK4 groups, respectively. To correlate functional antibody responses with *in vivo* protection, hamster sera were analyzed in Vero cell cytotoxicity assay. Hamsters vaccinated with rlipoA-RBD + B-rRBD again had generated good anti-TcdA and TcdB neutralizing antibody responses, the neutralization titers were 512 and 64, respectively (Table 1). These good anti-toxin antibody responses may correlate to better protection against a *C. difficile* spore challenge. Anti-toxin neutralization titers found in the hamster group vaccinated with A-rRBD + B-rRBD formulated with Pam3CSK4 was 32 and 8 against TcdA and TcdB, respectively. This low neutralization titers indicate that Pam3CSK4 did not provide a strong adjuvant effect. Current results also suggest that vaccine candidates containing 10 μg of both A-rRBD + B-rRBD formulated with alum provides partial protection (67 %).

The current studies have shown an interesting observation that a much less *C. difficile* colonized the selective TCCFA plates when the faecal pellets collected from the survived hamster groups vaccinated either with rlipoA-RBD + B-rRBD or A-rRBD + B-rRBD formulated with alum (Fig. 9b). These two groups have >60 % of survival rate (Fig. 9a). Around 30 to 50 CFU per 100 mg of faecal pellets were found in the selective TCCFA plates when the faecal pellets collected from these two survived hamster groups, but those obtained from other survived hamster groups were found to be significant higher, 300 to 5,000 CFU per 100 mg of faecal pellet (p < 0.01). It seems that the strong neutralizing antibody responses not only provide better protection against the toxicity of both toxins, but also help the host reduce the *C. difficile* bacteria counts.

A-rRBD and B-rRBD alone or formulated with alum did not provide full protection in the hamster challenge model; these results strongly indicate that an efficacious CDI vaccine requires the RBD from both TcdA and TcdB. This is consistent with previous report by Tian *et al*., [22] that a novel fusion protein containing the receptor binding domains of *C. difficile* toxin A and toxin B (3 × 100 μg of fusion protein formulated with alum adjuvant) elicited protective immunity against lethal toxin and spore challenge in preclinical efficacy models. Our current vaccine formulation containing 10 μg of rlipoA-RBD + 10 μg of B-rRBD consistently elicitstrong neutralizing antibody responses (Table 1) and protection (Fig 8a and 9a) against *C. difficile* spore challenge in the hamster model, and should be considered a strong vaccine candidate for CDI vaccine development and future clinical trials. Although the current studies have indicated that rlipoA-RBD can enhance immune responses through its TLR-2 agonist activity (intrinsic adjuvant property), further efforts are still required and should be pursued to optimize vaccine efficacy, including hamster challenge study with other BI/NAP1/027 hyper-virulent strains and a vaccination strategy for inducing rapid, strong and long lasting protective immunity in elderly and immuno-compromised individuals.

## Conclusion

*C. difficile* vaccine development is urgently needed to control the increasing incidence of hospital-acquired CDI that are responsible for rising medical costs. Although TcdA toxoid-based vaccines against CDI are currently in phase III clinical trials, recombinant antigens as vaccine candidates represent a new trend. Several studies have indicated that the neutralizing antibodies elicited by active immunization against clostridial toxins play very important roles in protection against infection and/or recurrence of CDI [13], [17], [35]. The present study, we have developed a cost-effective and efficacious recombinant subunit vaccine against CDI, using rlipoA-RBD as a rational design to contain a toll-like receptor 2 agonist (intrinsic adjuvant property) and achieved high yields in *E. coli* expression system. The purified rlipoA-RBD was characterized immunologically and found to have the following properties: (a) mice, hamsters and rabbits vaccinated with 3 μg of rlipoA-RBD produced strong antibody responses that could neutralize TcdA toxicity in the Vero cell cytotoxicity assay and the neutralization titer was comparable to those obtained from antisera immunized either with 10 μg of TcdA toxoid or 30 μg of A-rRBD; (b) rlipoA-RBD elicited immune responses and protected mice from TcdA challenge, but insignificant protection (10 to 20 %) against *C. difficile* spores challenge in the hamsters model; (c) only rlipoA-RBD formulated with B-rRBD consistently confers protection (90–100 %) in the hamsters challenge model; and (d) rlipoA-RBD was found to be 10-fold more potent than A-rRBD as an adjuvant to enhance immune responses against poor antigens such as ovalbumin. These results indicate that rlipoA-RBD formulated with B-rRBD would be an excellent vaccine candidate for preclinical studies and future clinical trials.

## Abbreviations

CDI: Clostridium difficile infection
TcdA: Clostridium difficile toxin A
TcdB: Clostridium difficile toxin B
A-rRBD: Recombinant C-terminal receptor binding domain of TcdA
B-rRBD: Recombinant C-terminal receptor binding domain of TcdB
rlipoA-RBD: Lipidated A-rRBD
CDAD: *C. difficile*-associated disease
RBD: Receptor binding domain
NHRI: National Health Research Institutes
LAL: Limulus amoebocyte lysate
IPTG: Isopropyl-β-D- thiogalacto-pyranoside
IMAC: Immobilized metal affinity chromatography
LPS: Lipopolysaccharide
OVA: Ovalbumin
ELISA: Enzyme-linked immunosorbent assay
NZW rabbit: New Zealand white rabbit
TFA: Taurocholatefructose-agar
TCCFA: Taurocholate-cefoxitin- cycloserinefructose-agar
CFU: Cell forming unit
DC: Dendritic cell
BMDC: Bone marrow-derived DC
MoGM-CSF: Recombinant granulocyte macrophage colony stimulating factor
LD_50_: The dose killing half of the subjects
MALDI-TOF: Matrix Assisted Laser Desorption/Ionization Time of Flight
